# Age-dependencies of the electroretinogram in healthy subjects

**DOI:** 10.1007/s10633-024-09991-w

**Published:** 2024-09-09

**Authors:** Ronja Jung, Melanie Kempf, Giulia Righetti, Fadi Nasser, Laura Kühlewein, Katarina Stingl, Krunoslav Stingl

**Affiliations:** 1 Center for Ophthalmology, University Eye Hospital, University of Tuebingen, Elfriede-Aulhorn-Str.7, 72076 Tuebingen, Germany; 2 Center for Rare Eye Diseases, University of Tuebingen, Tuebingen, Germany; 3 University Eye Hospital, University of Leipzig, Leipzig, Germany

**Keywords:** Aging, Cones, Electroretinogram, ISCEV standard protocols, On–off response, Rods

## Abstract

**Objective:**

The purpose of this study was to evaluate the age-dependency of amplitudes and implicit times in the electroretinograms (ERGs) of healthy individuals and provide clinicians and researchers with a reference for a variety of stimulus paradigms.

**Design and methods:**

Full-field electroretinography was conducted on 73 healthy participants aged 14–73 using an extended ISCEV standard protocol that included an additional 9 Hz flicker stimulus for assessing rod function and special paradigms for isolated On–Off and S-cone responses. Correlation coefficients and best-fit regression models for each parameter’s age-dependency were calculated.

**Results:**

Dark-adapted ERGs, in particular, displayed notable age-related alterations. The attenuation and delay of the b-wave with higher age were most significant in the dark-adapted, rod-driven 0.001 cd s/m^2^ flash ERG. The age-dependent reduction of the a-wave amplitude was strongest in the standard dark-adapted 3 cd s/m^2^ flash condition. Cone-driven, light-adapted responses to either flash or flicker stimuli displayed comparatively small alterations at higher age. S-cone function tended to diminish at an early age, but the effect was not significant in the whole population.

**Conclusion:**

The results suggest that rod and cone function decline at different rates with age, with rods being generally more affected by aging. Nonetheless, response amplitudes displayed a wide variability across the whole sample.

## Introduction

The electroretinogram (ERG) is the standard method for objectively evaluating retinal function. The standard full-field ERG (ffERG) that represents the summed potential of the entire neural retina has a biphasic waveform with two major components reflecting the initial hyperpolarization of photoreceptors (a-wave) and subsequent depolarization of bipolar cells (b-wave), respectively [29].

Various alterations of the ERG under specific pathological conditions have been described extensively in scientific literature. However, information on the changes in the ERG over the course of normal aging is scarce. Deficits in visual abilities generally become more frequent with advancing age. Likewise, the ERG is attenuated in the elderly population [5, 7, 8, 13, 15, 20]. Nevertheless, there is disagreement on the extent to which the individual retinal cell types and their associated ERG parameters are altered with age. Some older studies describe reductions of both a- and b-wave amplitudes [5]. Others mention reductions of rod but not cone response amplitudes in response to specific stimuli [13]. There is no complete set of reference values for age-related changes in the standard ffERG protocol and current parameters.

Hence, this study aimed to evaluate the age-dependent variations in the ffERG amplitudes and implicit times for an extended ISCEV (International Society for Clinical Electrophysiology of Vision) standard protocol. To this end, normative ffERG values were collected from 73 healthy participants of various ages.

## Methods

### Study design

The study was approved by the ethics committee of the University Hospital of Tuebingen. All volunteers were given detailed information about the methods and aims of the study before the examination, and their informed written consent was obtained. All subjects underwent a comprehensive ophthalmological examination, including best-corrected visual acuity (BCVA), intraocular pressure (IOP), indirect ophthalmoscopy, slit-lamp examination, and spectral domain optical coherence tomography. Subjects with any eye disease, cataracts, increased intraocular pressure, or visual acuity lower than 0.8 (decimal) were excluded (Table 1).

**Table 1:** Demographic information of the participants

Mean age ± standard deviation (years)	41 ± 17
Min. age (years)	14
Max. age (years)	73
Sample size	73 (27 M/46 F)

### Preparation and setup

A mydriatic containing tropicamide (0.5%) and phenylephrine hydrochloride (2.5%) was applied to the subject’s eyes to dilate the pupils to 8–9 mm. The participants’ facial skin was cleaned, and gold cup-electrodes, serving as reference and ground, were positioned on the temple region and forehead, respectively. The cornea was anesthetized with Oxybuprocainhydrochlorid 0.4% eye drops (Novesine^®^, OmniVision, Puchheim, Germany), and DTL fiber electrodes, serving as the active electrode, were placed on the corneal surface of the eye in the conjunctival bag. The right eye was recorded with a custom-made electrode (in-house production, comprising four filaments), while the left eye was measured with the commercially available DTL Diagnosys electrode to determine differences in ERG signals. For consistency, aging effects were analyzed using data from the right eye. The responses had a slightly larger amplitude with the custom-made electrode, but the age effects were generally similar in both eyes. Before the measurement, the electrodes’ impedance was tested and found to be smaller than 5 kΩ.

Light stimuli were presented in a spherical Ganzfeld bowl (ColorDome^®^ and Espion e2^®^/ e3^®^, Diagnosys LLC, Cambridge, UK). Raw signals were amplified and sent to a computer equipped with ERG analysis software provided by Diagnosys.

### Protocol

The examination included multiple ffERG measurements under dark-adapted (DA) and light-adapted (LA) conditions. Supplementing the six standard measurements recommended by ISCEV [29], additional flash ERGs with different intensities and other specialized ERG paradigms were performed, including a DA 9 Hz flicker stimulation, an On–Off ERG, and an S-cone stimulus. The stimulation parameters for all exams are provided in Table 2. LA ERGs were performed first after 10 min of light adaptation with a background luminance of 30 cd/m^2^. The subject was dark-adapted for at least 20 min prior to the DA measurements. As per convention, the amplitude of the a-wave was measured from baseline to the trough of the negative deflection, while the b-wave was measured from the trough of the a-wave to the peak.

**Table 2:** Parameters of the steps included in our ERG protocol

Step	Flash color	Flash strength (cd s/m^2^)*	Stimulus duration (ms)	Inter-stimulus interval (ms)	Background luminance (cd/m^2^) & color
LA 3.0 [SF]	White	3.0	4	1000	30.0 [White]
LA 31 Hz flicker	White	3.0	4	n.a.**	30.0 [White]
LA On–Off	White	0.32	240	500***	20.0 [White]
LA S-cone	Blue	0.2	10	1000	300.0 [Amber]
DA 0.001	White	0.001	4	10 000	n.a
DA 0.01	White	0.01	4	10 000	n.a
DA 9 Hz flicker	Blue	0.012 (scot)	10	15 000**	n.a
DA 0.1	White	0.1	4	10 000	n.a
DA 3.0 [SF]	White	3.0	4	15 000	n.a
DA 10.0 [HI]	White	10.0	4	15 000	n.a

Steps are listed in the order in which they were performed

The color and flash strength of background light are shown in parentheses. White light is defined by a color temperature of 6500 K. Blue is defined by a wavelength of 448 nm and amber by 590 nm

D, Dark-adapted; L, Light-adapted

^*^Luminance levels are given in phot units unless otherwise indicated

^**^For the 9 Hz flicker, ISI refers to the time between intervals of continuous stimulation. The data for the 31 Hz flicker was collected within a single interval of continuous stimulation, where possible

^***^Each flash was triggered manually. The indicated ISI refers to the smallest possible delay but was usually exceeded

### Flash ERGs

Cone-driven responses were obtained in LA state using the standard 3.0 cd s/m^2^ white flash stimulus. In DA state, five flash stimulations were applied sequentially with increasing time-integrated luminance levels of 0.001, 0.01, 0.1, 3.0, and 10.0 cd s/m^2^. Each stimulus had a duration of 4 ms. The light source had a color temperature of 6500 K. At least 5 sweeps were acquired per level.

### Flicker ERGs

The protocol included the standard 31 Hz flicker stimulation in LA state with a flash strength of 3.0 cd s/m^2^ and a duration of 4 ms. Additionally, a dark-adapted flicker 9 Hz ERG was performed to measure rod activity. This stimulus had a wavelength of 470 nm and a luminance of 3 scot cd/m^2^ (0.012 cd s/m^2^) with a duration of 10 ms. The 31 Hz flicker was applied continuously until 25 sweeps (0.25 s) with good signal quality were recorded. The 9 Hz flicker was split into three stimulation intervals with 6 sweeps (2.2 s) and an inter-stimulus interval of 15 s to maintain dark-adaptation. Sweeps that were disrupted by blink artifacts were automatically rejected. Raw traces were bandpass-filtered with cutoff frequencies of 1.25 and 30 Hz for the 9 Hz condition and 0.321 and 300 Hz for the 31 Hz condition. The amplitudes were calculated as the mean peak-to-peak difference of the averaged response. The implicit times were measured via cross-correlation between the averaged trace and a sinusoidal template.

### Oscillatory potentials

Oscillatory Potentials (OPs) were isolated from the DA 3.0 standard flash ERG using a bandpass filter with 75 and 300 Hz corner frequencies. Their power and implicit times were calculated using a continuous wavelet transformation as described by Righetti et al. [28].

### On–Off ERG

To isolate cone-driven On- and Off-channel ERGs, an achromatic long-duration stimulus with a luminance of 80 cd/m^2^ (0.32 cd s/m^2^) was presented against a rod-saturating white background of 20 cd/m^2^. The stimulus duration was increased to 240 ms (which exceeds the range recommended in the ISCEV guidelines; [32] to minimize the disruption of the c/d wave complex by blink-artifacts, which typically occur within the interval between On- and Off-deflections. Traces were averaged over at least 20 sweeps. Correlation analysis was conducted post-hoc to investigate the relationship between the mixed b-wave of the cone-driven short-flash ERG and the separated b- and d-waves of the On–Off ERG.

### S-cone ERG

The small contribution of short-wavelength (S) cones to the ERG is typically not measurable with the standard stimulus. For that purpose, the cones’ response was isolated using a 10 ms blue light stimulus with a luminance of 50.0 cd/m^2^ (0.2 cd s/m^2^) presented against an amber background (590 nm) of 300.0 cd/m^2^ to suppress long-wavelength (L) and middle-wavelength (M) cone responses. The S-cone activity thereby manifests as a second positive peak in the ERG, called s-wave, which is superimposed on a small b-wave elicited by L- and M-cones [2, 7, 11, 15]. Results were averaged over at least 20 sweeps.

### Statistics

**Fig. 1:**
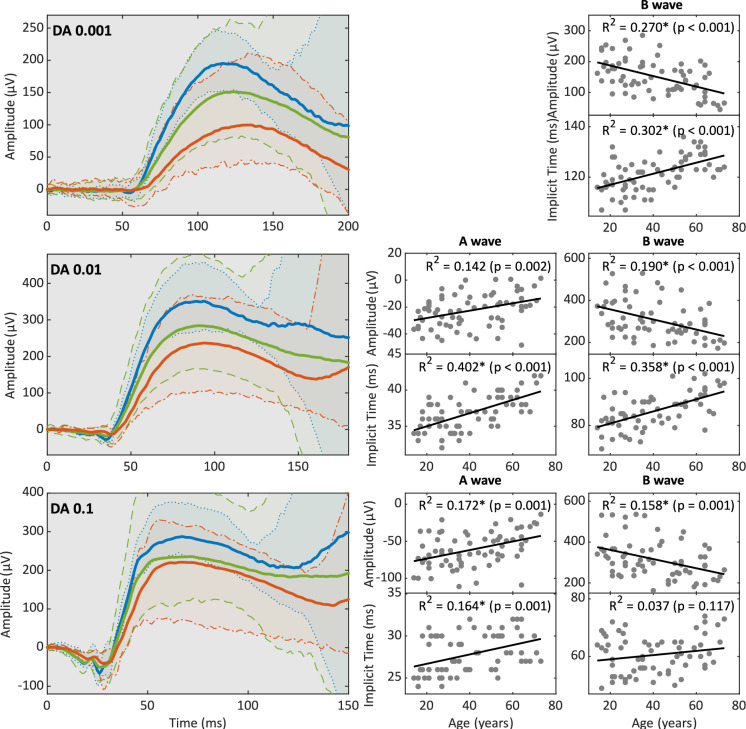
DA dim-flash ERGs. Shown here are the averaged ERG traces (lines) and 95% percentiles (shaded areas) of DA flash ERGs from healthy adults (21–60 years, green), in comparison to adolescents (< 20 years, blue), and older adults (> 60 years, red). The corresponding age distributions of all parameters are shown on the right

**Fig. 2:**
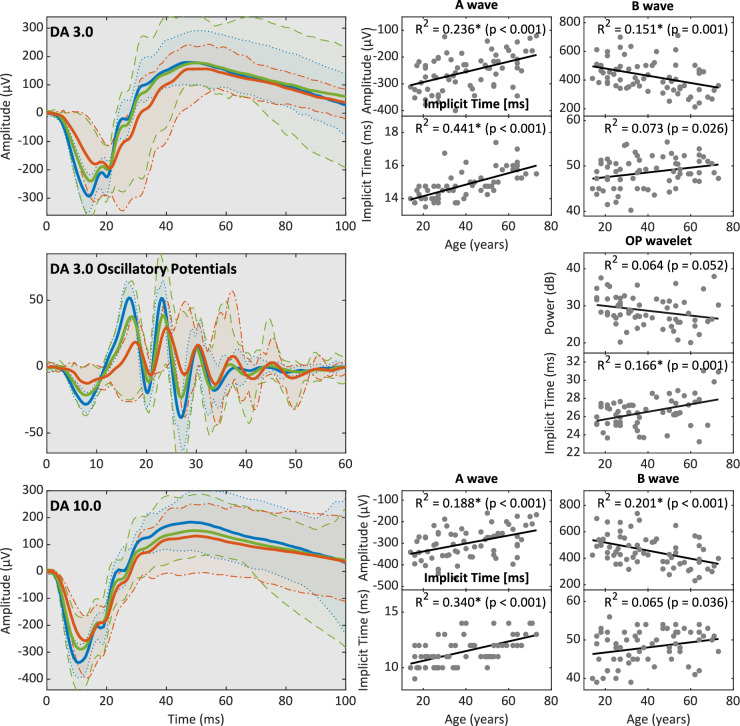
DA standard and high-intensity flash ERGs. Shown here are the averaged ERG traces (lines) and 95% percentiles (shaded areas) of the DA standard 3.0 and high-intensity flash 10.0 stimuli from healthy adults compared to adolescents and older adults (< 21-year-olds in blue, 21–60-year-olds in green, and > 60-year-olds in red). The corresponding age distribution of each parameter is shown on the right

**Fig. 3:**
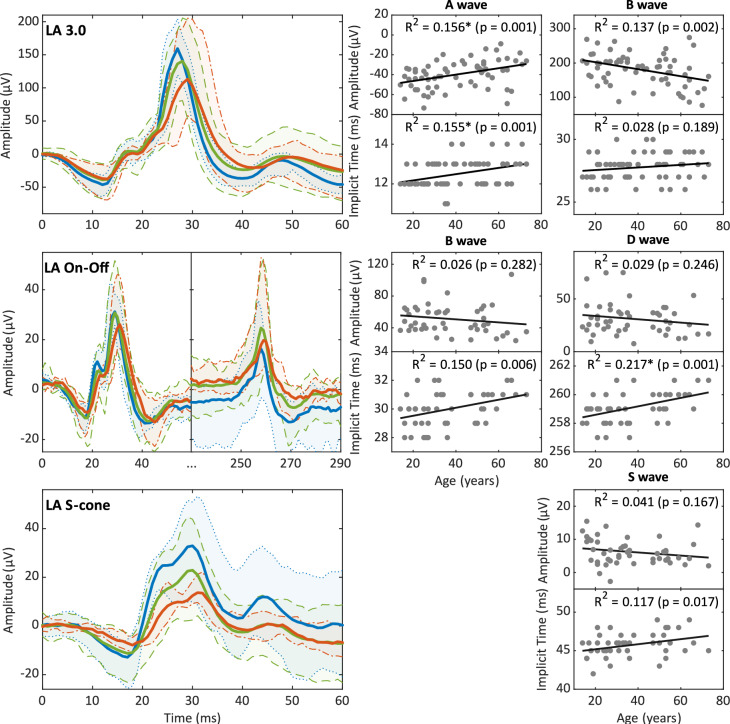
LA standard, long-duration flash, and s-cone ERGs. Shown here are the averaged ERG (lines) traces and 95% percentiles (shaded areas) from healthy adults compared to adolescents and older adults (< 21-year-olds in blue, 21–60-year-olds in green, and > 60-year-olds in red) for LA ERGs. The corresponding age distribution of each parameter is shown on the right

**Fig. 4:**
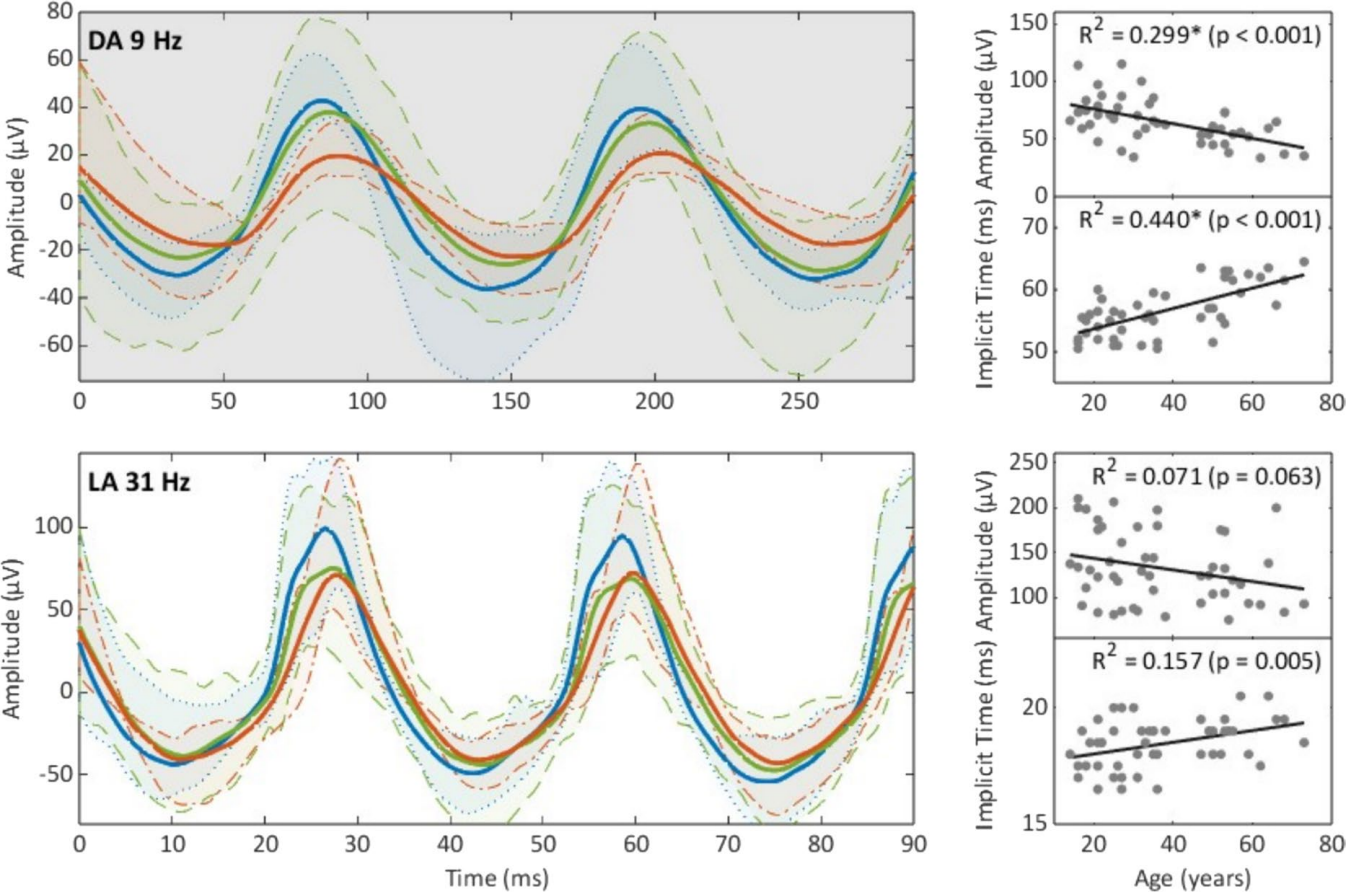
DA 9 Hz and LA 31 Hz flicker ERGs. Shown here are the averaged ERG traces (lines) and 95% percentiles (shaded areas) from healthy adults compared to adolescents and older adults (< 21-year-olds in blue, 21–60-year-olds in green, and > 60-year-olds in red) for two different flicker ERGs. The corresponding age distribution of each parameter is shown on the right

Age-related changes in the ERG parameters were statistically evaluated using IBM SPSS Statistics (SPSS Inc., Chicago, USA) and Matlab (The MathWorks, Inc., MATLAB, version 2022a). Age-dependencies for amplitudes and implicit times were calculated via pairwise linear correlation. The average change in parameters with higher age was calculated using the slope of the best-fit linear regression line. To highlight age effects on the raw ERG waveform, the averaged traces from normal adults (21–60 years, N = 51) are shown (Figs. 1, 2, 3, 4) along with the traces from our youngest participants (< 21 years, N = 8) and our oldest participants (> 60 years, N = 14). In addition, a correlation analysis among the different ERG parameters was conducted using Pearson’s correlation coefficient to determine inter-dependencies.

## Results

Many of the ERG parameters tested, including correlates of rod and cone and inner retinal function, showed significant age dependencies. Information on the distribution of the data and the results of the correlation analyses are summarized in Table 3.

**Table 3:** Age dependencies of different ERG stimuli

Step	Parameter		Mean	SD	95% IQR	Min	Max	R^2^	sign	Δ/10 years
LA 3.0	A	amp	− 40	14	− 69,− 14	− 9	− 74	0.156	< 0.001*	+ 3.2
		IT	12	1	11, 14	11	14	0.155	< 0.001*	+ 0.2
	B	amp	181	47	86, 268	76	275	0.137	0.002	− 10.3
		IT	28	1	26, 30	26	30	0.028	0.190	+ 0.1
LA 31 Hz flicker		amp	133	40	78, 207	75	210	0.071	0.063	− 6.4
		IT	18	1	17, 21	17	21	0.157	0.005	+ 0.3
LA On–Off	B	amp	51	20	25, 103	24	107	0.026	0.282	− 1.9
		IT	30	1	28, 32	28	32	0.150	0.006	+ 0.3
	D	amp	31	15	9, 75	8	75	0.029	0.246	− 1.6
		IT	259	1	257, 261	257	261	0.217	0.001*	+ 0.3
LA S-cone	S	amp	6	4	0, 15	0	15	0.041	0.167	− 0.5
		IT	46	2	43, 49	42	49	0.117	0.017	+ 0.3
DA 0.001	B	amp	151	57	58, 266	44	285	0.270	< 0.001*	− 17.1
		IT	121	7	108, 134	107	136	0.302	< 0.001*	+ 2.2
DA 0.01	A	amp	− 23	13	− 45,0	0	− 48	0.142	0.002	+ 2.7
		IT	37	2	33, 42	32	42	0.402	< 0.001*	+ 0.9
	B	amp	306	93	174, 522	111	529	0.190	< 0.001*	− 24.0
		IT	86	7	73, 101	70	102	0.360	< 0.001*	+ 2.6
DA 9 Hz flicker		amp	65	19	34,114	34	115	** 0.299**	< 0.001*	− 6.4
		IT	57	4	51, 64	51	65	** 0.440**	< 0.001*	+ 1.6
DA 0.1	A	amp	− 61	24	− 109, − 21	− 14	− 111	0.172	0.001*	+ 5.8
		IT	28	2	24, 32	24	32	0.164	0.001*	+ 0.6
	B	amp	314	97	165, 533	109	536	0.160	0.001*	− 22.5
		IT	61	6	50, 73	49	74	0.037	0.117	+ 0.8
DA 3.0	A	amp	− 254	67	− 375, − 125	− 120	− 400	0.236	< 0.001*	+ 19.2
		IT	15	1	14, 17	14	17	0.441	< 0.001*	+ 0.3
	B	amp	428	111	243, 686	214	710	0.151	0.001*	− 25.2
		IT	49	3	42, 55	40	55	0.073	0.026	+ 0.5
OPs		power	29	4	20, 38	20	38	0.064	0.052	− 0.6
		IT	27	2	24, 31	23	31	**0.167**	0.001*	+ 0.4
DA 10.0	A	amp	− 299	72	− 437, − 169	− 160	− 462	0.188	< 0.001*	+ 18.5
		IT	11	1	10, 14	9	14	0.340	< 0.001*	+ 0.4
	B									
		amp	456	116	257, 698	230	738	**0.201**	< 0.001*	−30.5
		IT	48	4	39, 54	39	56	0.065	0.036	+ 0.7

The table shows the distribution and age-related changes in ERG parameters in healthy individuals

Amplitudes are measured in µV, power in dB, implicit times (IT) in ms

IQR, inter-quantile range; IT, implicit time; SD, standard deviation

Asterisks indicate values below the Bonferroni-corrected significance threshold of *p* = 0.0016

The final sample included 73 healthy Caucasian individuals in the age range 14–73 (Table 1), including eight adolescents (14–20), 51 adults (21–60 years), and 14 people aged 61 and above. Differences in amplitudes and ITs between these groups are listed in Table 4.

**Table 4:** Distribution of ERG values across age groups

Step	Parameter		Mean ± SD & 95% IQR < 21	Mean ± SD & 95% IQR 21–60	Mean ± SD & 95% IQR > 60
LA 3.0	A	amp	− 46 ± 11 (− 65, − 34)	− 40 ± 13 (− 66, − 15)	− 35 ± 18 (− 69,− 9)
		IT	12 ± 0 (12, 13)	12 ± 1 (11, 14)	13 ± 1 (12, 14)
	B	amp	207 ± 38 (160, 269)	184 ± 41 (98, 260)	152 ± 62 (76,275)
		IT	27 ± 1 (26, 29)	28 ± 1 (26, 30)	28 ± 1 (26, 29)
LA 31 Hz Flicker		amp	151 ± 45 (91, 210)	130 ± 38 (76, 202)	121 ± 49 (84, 199)
		IT	18 ± 1 (17, 19)	18 ± 1 (17, 20)	19 ± 1 (18, 21)
LA On–Off	B	amp	50 ± 12 (37, 65)	52 ± 19 (25, 100)	46 ± 35 (24, 107)
		IT	29 ± 1 (28, 31)	30 ± 1 (28, 32)	31 ± 1 (31, 32)
	D	amp	30 ± 19 (10, 69)	32 ± 15 (9, 75)	25 ± 16 (13, 53)
		IT	259 ± 1 (258, 260)	259 ± 1 (257, 261)	260 ± 1 (259, 261)
LA S-cone	S	amp	10 ± 4 (4, 15)	5 ± 3 (0, 13)	6 ± 5 (2, 14)
		IT	45 + 1 (42, 46)	46 ± 1 (43, 49)	46 ± 2 (45, 49)
DA 0.001	B	amp	198 ± 43 (137, 254)	157 ± 50 (83, 274)	101 ± 55 (44, 202)
		IT	116 ± 4 (107, 121)	121 ± 7 (109, 135)	127 ± 5 (120, 134)
DA 0.01	A	amp	− 32 ± 7 (− 43, − 21)	− 23 ± 12 (− 44, 0)	− 15 ± 13 (− 48, 0)
		IT	35 ± 1 (33, 37)	37 ± 2 (33, 40)	40 ± 2 (37, 42)
	B	amp	373 ± 66 (285, 483)	313 ± 92 (191, 528)	244 ± 81 (111, 386)
		IT	81 ± 6 (70, 92)	85 ± 7 (74, 100)	93 ± 7 (78, 102)
DA 9 Hz Flicker		amp	76 ± 19 (59, 114)	65 ± 18 (36, 109)	46 ± 15 (34, 65)
		IT	53 ± 2 (51, 56)	56 ± 4 (51, 63)	62 ± 3 (58, 65)
DA 0.1	A	amp	− 74 ± 23 (− 100, − 37)	− 63 ± 22 (− 108, − 21)	− 46 ± 26 (− 109, − 14)
		IT	26 ± 2 (24, 30)	28 ± 2 (25, 32)	29 ± 2 (27, 32)
	B	amp	383 ± 75 (322, 532)	316 ± 98 (175, 534)	267 ± 80 (109, 385)
		IT	63 ± 6 (49, 69)	59 ± 6 (51, 71)	65 ± 7 (56, 74)
DA 3.0	A	amp	− 294 ± 51 (− 352, − 217)	− 258 ± 64 (− 383, − 136)	− 210 ± 69 (− 345, − 121)
		IT	14 ± 0 (14, 15)	15 ± 1 (14, 17)	16 ± 1 (15, 17)
	B	amp	476 ± 75 (383, 611)	437 ± 112 (262, 702)	365 ± 101 (214, 503)
		IT	47 ± 3 (44, 51)	49 ± 3 (41, 55)	50 ± 2 (47, 54)
OPs		power	33 ± 3 (28, 38)	28 ± 4 (20, 36)	28 ± 5 (22, 38)
		IT	26 ± 1 (24, 27)	26 ± 1 (24, 29)	28 ± 3 (23, 31)
DA 10.0	A	amp	− 340 ± 45 (− 395, − 270)	− 302 ± 71 (− 446, − 180)	− 259 ± 76 (− 397, − 167)
		IT	11 ± 1 (9, 12)	11 ± 1 (10, 14)	13 ± 1 (12, 14)
	B	amp	530 ± 85 (423, 700)	461 ± 114 (296, 704)	391 ± 112 (230, 569)
		IT	46 ± 6 (39, 53)	48 ± 4 (40, 55)	49 ± 5 (41, 54)

This table shows the differences in ERG parameters between healthy adults, adolescents, and older adults, corresponding to the traces shown in Figs. 1, 2, 3 and 4

IQR, inter-quantile range; IT, implicit time; SD, standard deviation

All participants had dilated pupils (median: 8.5 mm, 5% percentile: 7.5 mm, 95% percentile: 9.0 mm), and pupil size did not decline with age (rho = − 0.002, *p* = 0.986, Spearman). Pupil size had no significant effect on any of the parameters. The participants’ median BCVA was 1.25. Visual acuity was significantly correlated to age (rho = − 0.416, *p* < 0.001, Spearman).

### Flash ERGs

The dim DA 0.001, 0.01, and 0.1 flash stimuli elicit rod-driven responses (Fig. 1). Despite high inter-individual variabilities, significant reductions of the b-wave amplitude with higher age (ranging from 17 to 24 µV/ 10 years) were detected at all three stimulus intensities. Older individuals also exhibited significantly longer b-wave implicit times in the DA 0.001 and DA 0.01 conditions. The rod-driven a-wave was detectable in the DA 0.01 and 0.1 ERGs and displayed a gradual reduction in amplitude and longer implicit times with higher age.

The DA 3.0 and DA 10.0 flash are measures of combined rod and cone activity and have pronounced a- and b-waves. A-wave amplitudes declined at similar rates in the DA 3.0 and DA 10.0 flash condition, by about 20 µV/ 10 years. The amplitude reduction was accompanied by a small but significant increase of the implicit time by 0.3–0.4 ms/10 years. The corresponding b-wave amplitudes diminished by 25–30 µV/ 10 years. The implicit times (ITs) tended to increase with higher age, but this difference was not significant overall.

The LA 3.0 ERG parameters displayed comparatively lower age-dependencies (Fig. 3). A-wave amplitudes declined significantly by approximately 3 µV/ 10 years, accompanied by a small but significant increase in the IT of 0.2 ms. The b-wave amplitude decreased by 10 µV. Its IT remained very stable across the whole sample.

### Flicker ERG

Age affected the two flicker ERGs to different extents (Fig. 4). The amplitude of the DA 9 Hz ERG, a measure of rod function, significantly diminished by ca. 6 µV/ 10 years. The IT increased significantly by 1.6 ms/ 10 years (R^2^ = 0.440, *p* < 0.001). The amplitude and IT of the cone-driven LA 31 Hz flicker were not significantly correlated with age.

### Oscillatory potentials

Oscillatory potentials were extracted from the DA 3.0 flash ERG. The power of the Ops displayed a high variability among subjects and was not significantly correlated to age (Fig. 3). The corresponding ITs increased by 0.4 ms/ 10 years (R^2^ = 0.167, *p* = 0.001).

### On–off ERG

The LA On–Off ERG uses a long-duration light stimulus (Sustar et al. 2018a), producing an initial b-wave at light onset, which corresponds to the On-response, and a second d-wave at stimulus offset, which represents the isolated Off-response. Although the b- and d-wave amplitudes tended to decline with higher age by almost 2 µV/ 10 years, these effects were not significant overall (Fig. 3). However, there were significant age-related prolongations of the b- and d-wave implicit times of almost 2 ms/ 10 years.

The On- and Off-responses were significantly correlated with each other in amplitudes and implicit times. Additionally, both the b- and d-wave were correlated to the b-wave in the standard LA 3.0 cd s/m^2^ ERG.

### S-cone ERG

**Fig. 5:**
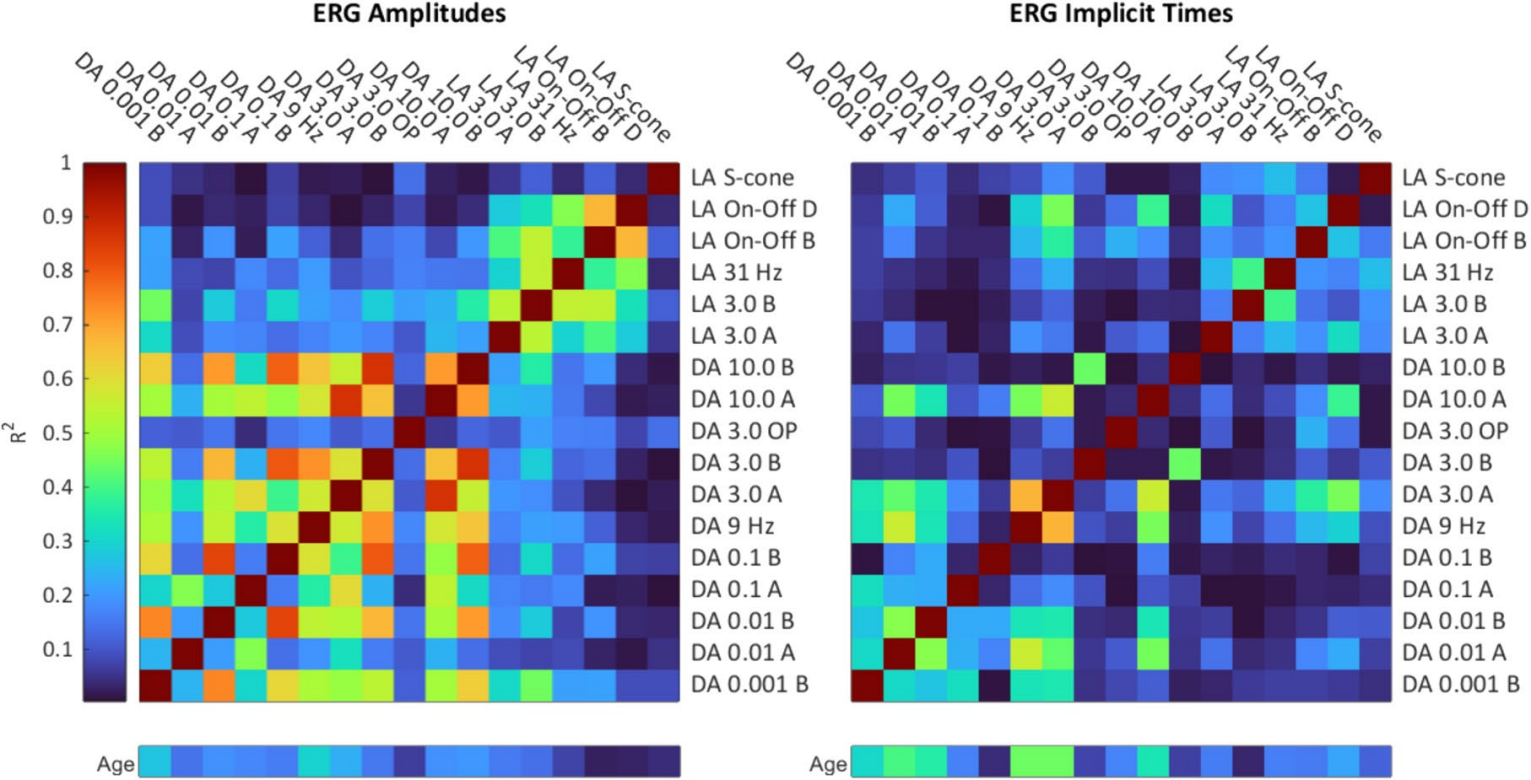
Dependencies between parameters of different ERG steps (overview). The heat-map displays the R^2^ values of the pairwise correlations between the individual parameters, i.e., red indicates high dependency while blue hues indicate low dependency between the two parameters labelled along the x- and y-axes, respectively. Age-dependencies (R^2^ values) for each parameter are indicated beneath the main plot

The s-wave showed no significant age dependence across the sample (Fig. 3), although the s-wave amplitudes of adolescents were 4 µV bigger on average than older age groups (Fig. 5). The s-wave IT increased slightly by 0.3 ms/10 years.

### Correlations between ERG parameters

Some ERG stimuli are designed to assess the isolated function of rods and cones. In other paradigms, their relative contribution to the final response is less well-defined. To explore the interdependencies between different paradigms and identify potential redundancies, a correlation analysis between all ERG parameters was performed (i.e., between a- and b-amplitudes and their corresponding ITs).

The R^2^-values are visualized in the heatmap in Fig. 5. The results show strong dependencies between almost all amplitudes of the DA ERG responses, which represent pure rod or combined rod/ cone channel function. B-wave amplitudes are usually more strongly correlated to one another than to their matching a-waves, with R^2^ values ranging from 0.538 (between the DA 0.001 and DA 3.0 b-wave amplitudes) to 0.872 (between the DA 3.0 and DA 10.0 b-amplitudes).

The amplitude of the DA 9 Hz flicker response was highly correlated to the b-waves of other DA steps, particularly the mixed response of the DA 3.0 ERG. Moreover, its IT displayed high dependency to the DA 3.0 a-wave implicit time (R^2^ = 0.675).

LA parameters of cone function (e.g., 3.0 flash ERG) were uncorrelated with parameters of DA steps. The 31 Hz flicker response displayed the highest dependency with the standard 3.0 cone-driven b-wave (R^2^ = 0.549), although this value was lower than the high dependencies between DA parameters (e.g., the dependency between the 9 Hz flicker and DA 3.0 b-wave amplitudes was R^2^ = 0.728). The 31 Hz flicker amplitude displayed higher dependency with the Off-channel D-wave (R^2^ = 0.464) than the isolated On-channel response (R^2^ = 0.381). Furthermore, the b- and d-wave amplitudes of the On–Off ERG were strongly correlated. The S-cone response, conversely, did not correlate strongly with other measures from the LA steps. Amplitude was best correlated with the power of the OP (R^2^ = 0.138). Its IT displayed a comparatively higher dependency with the 31 Hz flicker (R^2^ = 0.260).

## Discussion

The ERG waveform can be altered by various pathologic conditions in specific ways. For the correct interpretation of clinical ERGs and the accurate assessment of disease stages, age-matched reference values for the healthy retina should be established for all stimuli. The age-related variations of ERG parameters have been investigated in previous studies [5], but some of them used customized protocols [22] that may not be fully comparable with clinical measurements based on ISCEV standards [4, 8, 13]. In other cases, the analysis was restricted to individual parameters of the ERG waveform [7, 15, 20]. The purpose of this study was to provide a local database for age-dependencies in the ISCEV standard ERG protocol (including commonly used supplementary paradigms) and reveal interdependencies between these responses to aid fellow researchers and clinicians in predicting possible covariates in their experiments and long-term changes in clinical assessments.

### Age effect and correlations between measures

Several amplitudes and ITs of different steps have been found to change with the subjects’ age. In general, parameters of rod functions were more severely affected by age than parameters of cone functions. These changes likely result from an interplay between various processes of retinal and non-retinal origin that occur during normal aging, which are discussed in the following sections.

### Photoreceptor function

The a-wave functionally depends on the transparency of the optical media, the number of responding photoreceptors, and their metabolic supply through the RPE and Bruch’s membrane. In this study, the linear regression models for the age-dependency of the a-wave reveal amplitude reductions of 27% –49% and accompanying delays in implicit times of 6%–20% at age 70 compared to age 20. Similar observations have been made in prior studies in healthy individuals [4, 8]. To some extent, the change in amplitude can be attributed to the age-related yellowing of the lens, which causes a reduction of the effective retinal illuminance, especially in the short-wavelength range [6]. However, the specific magnitude of the changes was found to depend on the stimulus strength and thus on the type of photoreceptor. Specifically, the rod-mediated a-wave (0.01 and 0.1 cd m^2^/s conditions) declined on average by 44% in 50 years, whereas mixed and pure cone responses decreased by 32%, according to our models. Similar differences between rods and cones were observed for the corresponding ITs. These differences suggest the attenuation of the a-wave is influenced by cell-specific factors. This interpretation is supported by previous psychophysical findings, such as the delay in the human dark-adaptation curve [19] and the selective loss of rod sensitivity in older individuals [30], which could not be explained by pre-retinal factors alone.

The slow decline of rod density with advancing age is a factor that may explain the more pronounced amplitude reduction of the rod-driven a-wave [9, 14]. Cones, in contrast, are assumed to be well preserved throughout life [9, 14]. Nevertheless, a reduction in the number of photoreceptors does not explain the increasing a-wave IT observed across different light intensities.

Exploring alternative reasons for these changes has led to the consideration of various metabolic or functional mechanisms, including changes in photopigment optical density [18, 23], dysfunction of the retinal pigment epithelium [12, 21, 31], and limited vitamin A supply due to the thickening of Bruch’s membrane [17, 24, 27]. The S-cone is the photoreceptor type most susceptible to metabolic or ischemic damage [16]. For this reason, the S-cone ERG examination is sometimes used as an indicator for the progression of certain systemic disorders, such as retinopathic diabetes [16, 25, 34]. In this study, S-cones displayed a sudden amplitude reduction at the end of adolescence (Fig. 3, left column) rather than a gradual attenuation (as one would expect from the yellowing of the lens) in response to a blue-flash stimulus. This finding might point to a transition in retinal function in early adulthood.

Overall, our results suggest that photoreceptors are influenced by several retinal and preretinal factors. Although our study cannot provide a clear explanation of how each of these mechanisms modulates the electrophysiological response, the results provide a basis for future studies aimed at disentangling the underlying factors.

### Inner retinal function

The b-wave is a function of inner retina processing and input from the outer retina. Compared to the severe attenuation of the preceding a-wave, the b-wave displayed relatively smaller reductions across DA and LA conditions. For instance, the b-wave amplitude of the rod-mediated 0.01 cd m^2^/s ERG declined by only 34% in 50 years, while the a-wave declined by 49%. A similar difference was found in the LA standard flash condition, which displayed a b-wave reduction of 25% and an a-wave reduction of 35% at age 70 compared to age 20. Similarly, the change in the b-wave IT was comparatively smaller than the change in the preceding a-wave. These differences indicate high preservation of the inner retina in higher ages and hint at the existence of compensatory mechanisms to counteract signal attenuation in the outer retina.

Oscillatory potentials (OPs) are small wavelets superimposed on the rising phase of the b-wave. Though the exact cause of OPs is unknown, they are assumed to arise through interactions between bipolar, amacrine, and retinal ganglion cells in the inner retina [35]. In this study, we found a comparatively small reduction in the power of OPs, which was accompanied by a small but significant delay. The parameters were calculated and analyzed as a whole using continuous wavelet transformation. This approach has previously revealed a significant reduction in OP power in achromatopsia patients, suggesting that OPs are linked to cone activity [28]. However, we found no strong correlations between OPs and other ERG parameters to corroborate these findings. A more detailed investigation of the individual OP components might provide further information on the underlying mechanisms. For example, Kergoat et al. [22] found that single wavelets were differentially affected by age [22], suggesting that they are each involved in different processes.

### Dependency between parameters

In the second part of this study, we analyzed the interdependencies between parameters of different steps. Generally, correlations between inner and outer retinal responses from the same step were relatively small, showing that the b-wave’s shape is determined by photoreceptor inputs and inner retinal processing mechanisms [10, 26].

Furthermore, the results suggest that DA flash ERGs across different stimulus intensities provide largely redundant information about the inner retina, as evidenced by the high correlations amongst their b-waves. The a- and b-wave of the LA 3.0 flash ERG were only weakly correlated with the mixed b-wave of the DA 3.0 and DA 10.0 ERGs compared to responses to lower flash intensities (DA 0.001–0.1), suggesting that these responses are still dominated by rods [3]. The comparatively low dependency between the DA a-waves (e.g., the DA 0.01 and 0.1 a-waves) can be explained by their small amplitudes and low signal-to-noise ratio, making them comparatively less reliable as parameters for rod responses.

Conversely, the 9 Hz flicker displayed a strong correlation with other DA b-wave amplitudes (e.g., the DA 0.1 and 3.0 b-waves) and a high correlation with a-wave ITs (e.g., the DA 0.01 and 3.0 a-waves) across different stimulus intensities and may thus be a suitable age-specific alternative to the standard DA 0.01 flash ERG for assessing rod function.

The relationship between the 31 Hz flicker and the standard 3.0 flash ERG was unexpectedly low in absolute values compared to rod function parameters. This finding is unlikely to be due to differences in the methods used to evaluate flicker parameters, considering the high dependencies of the flickers at 9 Hz. Instead, the finding suggests that the 31 Hz oscillation is more than just a concatenation of standard b-waves and that cones and on- and off-bipolar cells react differently depending on the temporal dynamics of the applied stimulus. In fact, data from an animal study indicate that (in mice) the 30 Hz flicker may be dominated by off-channel activity, while low-frequency responses (including the standard flash) are primarily mediated by on-bipolar cells [33]. In line with this model, we found that the 31 Hz flicker amplitude was slightly better correlated to the d-wave than the b-wave in the on–off ERG.

### Limitations

This study has limitations that need to be considered in the analysis of clinical ERG data.

An important factor not included in this study but known to have a significant impact on ERG amplitudes is fundus pigmentation: Eyes with higher melanin concentrations in the RPE and choroidal layers reflect less light back to the photoreceptors, resulting in weaker ERG signals. Because fundus pigmentation directly correlates with eye color, individuals with brown eyes generally have lower amplitudes and slightly longer implicit times than blue-eyed individuals [1]. All participants in the present study were Caucasian, including many blue-eyed individuals. Therefore, normative values for other countries and ethnic groups will be slightly smaller than reported here.

Another potential limitation of our study is the gender imbalance among the participants. Our sample included 46 females and 27 males. Both groups had a similar age distribution. On average, though, women had larger amplitudes and slightly shorter implicit times than males. The b-wave amplitude of the DA 10.0 b-wave, for instance, was 53 µV larger in women (475 ± 113 µV) compared to men (422 ± 111 µV). Furthermore, women usually displayed stronger absolute and relative changes in ERG amplitudes and implicit times.

Additionally, differences in the equipment used may limit the comparability of the results to external clinical data. Above all, the quality and strength of the signals depend heavily on the type of electrodes used. In this study, we used a custom-made DTL electrode. To assess differences in ERG signals between our electrode and commercially available ones, participants’ left eyes were measured with the DTL Diagnosys electrode. On average, our electrode produced slightly larger signals—for example, the average inter-ocular difference in b-waves varied between 5 µV in the DA 0.001 ERG (DTL Tuebingen: 151 ± 57 µV for vs DTL Diagnosys: 152 ± 60 µV) and 45 µV in the DA 10.0 ERG (456 ± 116 µV vs 409 ± 123 µV). In most cases, these differences were not statistically significant and, overall, the absolute decrease in ERG amplitudes was slightly lower for the left eye. Furthermore, the general trends for age-dependencies were the same for both electrode types.

Ultimately, the data presented here cannot be generalized to all subject groups and setups. Nevertheless, our results may serve as a reference for age-dependent effects in ERG measurements that will hopefully help researchers and clinicians design and interpret their own experiments and clinical measurements.

## Conclusion

The ERG of healthy individuals undergoes significant changes with advancing age. Accordingly, patients’ age should be considered when evaluating clinical ERGs. Overall, the weakening of ERG signals can be caused by changes in the optical media and metabolic alterations in subretinal layers. However, the comparatively stronger age effects in the rod-driven ERG point to selective alterations of rod function or number in the healthy retina. Cone responses, as measured via light-adapted flash and 31 Hz flicker ERGs, were found to be relatively stable. Dark-adapted and light-adapted responses do not closely correlate, indicating different cellular causes.

## Abbreviations

amp: Amplitude
D: Dark-adapted
ERG: Electroretinogram/electroretinography
ffERG: Full-field electroretinogram/electroretinography
ISCEV: International Society for Clinical Electrophysiology of Vision
ISI: Inter-stimulus interval
IT: Implicit time
L: Light-adapted
OP: Oscillatory potentials
SD: Standard deviation

## Declarations

### Human and animals Right

The study was approved by the ethics committee of the University of Tübingen. All procedures performed in studies involving human participants were in accordance with the ethical standards of the University of Tübingen and with the 1964 Helsinki declaration and its later amendments. This article does not contain any studies with animals performed by any of the authors.
